# Leveraging large-scale multi-omics evidences to identify therapeutic targets from genome-wide association studies

**DOI:** 10.1186/s12864-024-10971-2

**Published:** 2024-11-19

**Authors:** Samuel Lessard, Michael Chao, Kadri Reis, FinnGen, Estonian Biobank Research Team, Mathieu Beauvais, Deepak K. Rajpal, Jennifer Sloane, Priit Palta, Katherine Klinger, Emanuele de Rinaldis, Khader Shameer, Clément Chatelain

**Affiliations:** 1 Precision Medicine & Computational Biology, Sanofi, Cambridge, MA USA; 2 Estonian Genome Centre, Institute of Genomics, University of Tartu, Tartu, Estonia; 3 Digital R&D Data & Computational Sciences, Sanofi, Gentilly, France; 4 Translational Sciences, Sanofi, Framingham, MA USA; 5 Pre-Clinical and Translational Sciences, Takeda, MA USA; 6 Immunology & Inflammation Development, Sanofi, Cambridge, MA USA; 7 Genetics Research, Sanofi, Cambridge, MA USA

**Keywords:** Genome-wide association study, Molecular quantitative trait loci, Causal inference, Therapeutic targets, Interleukin 6, Polymyalgia rheumatica, Mendelian randomization

## Abstract

**Background:**

Therapeutic targets supported by genetic evidence from genome-wide association studies (GWAS) show higher probability of success in clinical trials. GWAS is a powerful approach to identify links between genetic variants and phenotypic variation; however, identifying the genes driving associations identified in GWAS remains challenging. Integration of molecular quantitative trait loci (molQTL) such as expression QTL (eQTL) using mendelian randomization (MR) and colocalization analyses can help with the identification of causal genes. Careful interpretation remains warranted because eQTL can affect the expression of multiple genes within the same locus.

**Methods:**

We used a combination of genomic features that include variant annotation, activity-by-contact maps, MR, and colocalization with molQTL to prioritize causal genes across 4,611 disease GWAS and meta-analyses from biobank studies, namely FinnGen, Estonian Biobank and UK Biobank.

**Results:**

Genes identified using this approach are enriched for gold standard causal genes and capture known biological links between disease genetics and biology. In addition, we find that eQTL colocalizing with GWAS are statistically enriched for corresponding disease-relevant tissues. We show that predicted directionality from MR is generally consistent with matched drug mechanism of actions (> 85% for approved drugs). Compared to the nearest gene mapping method, genes supported by multi-omics evidences displayed higher enrichment in approved therapeutic targets (risk ratio 1.75 vs. 2.58 for genes with the highest level of support). Finally, using this approach, we detected anassociation between the IL6 receptor signal transduction gene *IL6ST* and polymyalgia rheumatica, an indication for which sarilumab, a monoclonal antibody against IL-6, has been recently approved.

**Conclusions:**

Combining variant annotation, activity-by-contact maps, and molQTL increases performance to identify causal genes, while informing on directionality which can be translated to successful target identification and drug development.

**Supplementary Information:**

The online version contains supplementary material available at 10.1186/s12864-024-10971-2.

## Background

Genome-wide associations studies (GWAS) have been successful in identifying genes associated with traits, diseases, and molecular phenotypes [1, 2]. Discoveries from GWAS have increased substantially over the years due to low cost of genomic profiling technologies, an increased number of studies, larger cohorts, and meta-analyses, as well as the formation of deeply phenotyped datasets [3]. The latter include large-scale biobank projects such as UK Biobank (UKB) [4, 5], Estonian Biobank [6], and FinnGen [7]. As an example, the UK Biobank alone has contributed to over 3,200 publications (https://www.ukbiobank.ac.uk/enable-your-research/publications), and the FinnGen project is set to increase the number of discoveries emerging from rare variants enriched in the Finnish population [7]. Similarly, the Estonian Biobank, with its extensive dataset, has enhanced rare and low-frequency genetic variation discoveries [8–10]. 

Discoveries from genetic studies provide a highly valuable resource for drug discoveries. For example, therapeutic targets with genetic support are > 2 times more likely to succeed in clinical trials [11, 12]. A notable example is the association between a loss-of-function missense variant in *IL23R* gene and Crohn’s disease, suggesting that IL-23 blockage could be beneficial [13–16]. Drugs targeting the IL-23 receptor including Ustekinumab and Risankizumab have recently been approved by the FDA for the treatment of Crohn’s disease following successful clinical trials [17–19]. Other notable examples of targets supported by GWAS include *IL6R* for rheumatoid arthritis (Sarilumab, Tocilizumab) and *HMGCR* for high levels of low-density lipoprotein (statins) [20, 21]. 

While these examples clearly show that genetic disease associations provide important information for drug development, it remains a challenge to accurately assign causal genes driving disease risk from GWAS as most variants identified in GWAS fall in non-coding regions of the genome [22–24]. While it’s been observed that the nearest gene often is the causal gene, this is not a guarantee as genetic variants can influence traits over large genomic distances [25]. In addition, this observation may be biased towards genes that have been well-characterized because they fall at the center of genetic association signals [26]. 

Several approaches have been used to predict causal genes, including selecting the nearest gene, variant pathogenicity predictions, epigenetic interactions, and integration of molecular quantitative trait loci (molQTL) such as expression QTL (eQTL). Mendelian randomization (MR) integrating GWAS and molQTL can help identify causal relationships while informing on directionality but may be confounded due to linkage disequilibrium (LD) [27–29]. On the other hand, colocalization approaches can be used to detect whether molQTL and GWAS signals share a common causal variant in a specific locus [30, 31]. While colocalization approaches can link genetic variation to changes in gene expression in specific tissue or cell-type contexts, they also tend to be pleiotropic and often impact the expression of multiple genes within the same locus [26, 32, 33]. They can also impact expression across multiple tissues and cell types, decreasing their utility to identify pathogenic cell types [32, 34, 35]. In addition, a large fraction of GWAS loci don’t show eQTL signals, potentially due to the unavailability of data for relevant cell types or specific biological contexts or variants affecting disease risk due to different mechanisms such as splicing [32, 36, 37]. Despite these challenges, eQTL has successfully been used to identify causal genes [38, 39]. In addition, recent prioritization approaches such as the Locus to Gene (L2G) scores from Open Targets have shown that incorporating molecular trait information does increase performance to identify relevant genes [26]. 

Here, we sought to use currently available eQTL information to identify disease relevant genes in the context of drug discovery. We first derived a simple approach to prioritize causal genes based on MR [40], eQTL colocalization [31], activity-by-contact (ABC) enhancer-promoter interactions [41], and variant annotations [42]. We used this combinatorial approach as a way to mitigate the pleiotropic effect of eQTL while retaining important information about directionality. We show that this approach enriches for gold standard genes [26] and captures known target biology. In addition, genes prioritized by this approach are enriched for drug targets with successful clinical trials, and directionality inferred by MR or coding variants recapitulate drug mechanisms of action (MoA). Finally, we show that this approach can be used to identify drug indication expansion opportunities using genes related to the IL6 receptor as a case study and identify an association between *IL6ST* and polymyalgia rheumatica.

## Methods

### Estonian Biobank GWAS

The Estonian Biobank (EstBB) is a population-based biobank with 200k participants. The 198k data freeze was used for the analyses described here. All biobank participants have signed a broad informed consent form.

All EstBB participants have been genotyped at the Core Genotyping Lab of the Institute of Genomics, University of Tartu, using Illumina Global Screening Array v1.0 and v2.0. Samples were genotyped and PLINK format files were created using Illumina GenomeStudio v2.0.4. Individuals were excluded from the analysis if their call-rate was < 95% or if sex defined based on heterozygosity of X chromosome did not match sex in phenotype data. Before phasing and imputation, variants were filtered by call-rate < 95%, HWE *p* value < 1e-4 (autosomal variants only), and minor allele frequency < 1%. Variant positions were updated to b37 and all variants were changed to be from TOP strand using GSAMD-24v1-0_20011747_A1-b37.strand.RefAlt.zip files from https://www.well.ox.ac.uk/~wrayner/strand/ webpage. Chip data pre-phasing was done using Eagle v2.3 software [43] (number of conditioning haplotypes Eagle uses when phasing each sample was set to:–Kpbwt = 20000) and imputation was done using Beagle v.28 Sep18.7932 [44] with effective population size ne = 20,000. Population specific imputation reference panel of 2297 WGS samples was used [44]. 

### FinnGen

The FinnGen study (https://www.finngen.fi/en) was described previously [7]. The study is a public-private research project that combines genetic and healthcare data of over 500,000 Finns. The objective of the FinnGen study is to identify novel medical and therapeutical insight into human diseases. It is a pre-competitive partnership of Finnish biobanks, universities and university hospitals, international pharmaceutical industry partners, and Finnish biobank cooperative (FINBB). A full list of FinnGen partners is published here: https://www.finngen.fi/en/partners.

### Disease GWAS processing

We retrieved GWAS results from FinnGen release 10 (R10), UK Biobank pan-ancestry analysis [45], and a meta-analyses between FinnGen, UK Biobank, and Estonian biobank. For simplicity, we use the term GWAS to refer to both single study GWAS and meta-analyses throughout the manuscript. In total, we included 4,611 GWAS with at least one variant with *P* < 1 × 10^−6^. When appropriate, we lifted over variants from hg38 to hg19 using the liftOver R package [46]. Variant with a minor allele frequency (MAF) < 0.0001 were excluded from the analysis. For each GWAS, we considered genes located within 250 kb of a variant with *P* < 1 × 10^−6^ for further analysis. For gold standard and clinical trial enrichment analyses (described below), only genome-wide significant loci were included (*P* < 5 × 10^−8^). We excluded the human leukocyte antigen (HLA) region in all analyses.

### Disease EFO mapping

In order to perform semantic integration of genetic data and clinical trial data, the ontological system Experimental Factor Ontology (EFO) was used. We used the EFO to map traits to their corresponding EFO categories and when multiple EFO terms could be mapped to the same trait, we assigned the trait to each possible term. We used the EFO version 3.52.0 (https://www.ebi.ac.uk/efo/).

### Variant annotation

We used variant effect predictor (VEP v102) [42] to annotate the impact of variants with the following options: --everything --offline --check_existing --distance 250,000. Coding variants were defined as those impacting protein coding transcript annotated as missense variant or predicted to have “high” impact, including stop gain, splice-site, and frameshift variants. We also retrieved pathogenicity predictions for missense variants from ProtVar [47], considering conservation, structure stability predictions, and EVE [48] and ESM1b scores [49]. We defined pathogenic variants as those with “high” impact, predicted to be pathogenic, destabilizing, or in a conserved region. In addition, we linked non-coding variants to genes using activity-by-contact (ABC) maps [41]. ABC scores represent the contribution of an enhancer to the regulation of genes, measured by multiplying the estimates of enhancer activity and three-dimensional contact frequencies between enhancers and promoters. ABCmax refers to variant-gene pairs with the highest ABC score. We also retrieved disease mutations from the Human Gene Mutation Database (HGMD) (licensed from Qiagen, Maryland) [50]. We annotated all variants with *P* < 1 × 10^−6^ and within 5 orders of magnitude of the lead variant at the locus.

### Mendelian randomization & colocalization

We performed transcriptome wide MR using the R package TwoSampleMR [40]. When more than one instrument was present, we used the inverse variant weighted approach, otherwise we used the Wald Ratio approach. We considered the following exposures: protein quantitative trait loci (pQTL) from Sun et al. [51], and eQTL from Blueprint [52], eQTLGen [53] and other datasets from the EBI eQTL catalogue [53–77]. In total, 110 molQTL from 26 studies were included. For each of those studies, we excluded variants with a MAF < 1%. We clumped variants using PLINK [78] using the options –clump-p1 1 –clump-p2 1 –clump-r2 0.01 – clump-kb 10,000 and using the European ancestry subset of the 1000 Genomes Project phase 3 data as reference [79]. We included all genes within 250 kb of a GWAS variant with *P* < 1 × 10^−6^. For each QTL, independent variants with *P* < 1 × 10^−4^ were used as instruments. For genes with significant MR results (false discovery rate < 0.05), we also performed colocalization analysis using COLOC [31] in order to account for pleiotropy due to linkage, using a region of 250 kb each side of the local lead GWAS variant. Harmonization between molQTL and GWAS datasets was performed using the harmonise_data function in the TwoSampleMR package [40]. Only autosomes were included in this analysis.

### Causal gene prioritization

We prioritized genes as putatively causal using a combination of evidence including MR, colocalization H4 posterior probabilities (PP) with molQTL, presence of an associated pathogenic variant or other coding variants, distance to lead variant, and enhancer-promoter ABC scores [41]. Specifically, we ranked genes as follow:

**Table:** 

Rank	Criteria
**Very High**	Lead pathogenic variant; Or Colocalization (H4 PP > 80%) with molQTL of the target gene in > 2 dataset; and maximum ABC score for a regulatory element overlapping the lead variant
**High**	Lead coding variant; Or Associated (*P* < 1 × 10^−6^) pathogenic variant; Or Colocalization (H4 PP > 80%) with molQTL of the target gene in > 2 dataset and maximum ABC score for an associated variant overlapping a regulatory element (*P* < 1 × 10^−6^) Or Colocalization (H4 PP > 80%) with molQTL of the target gene in one dataset; and maximum ABC score for a regulatory element overlapping the lead variant
**Moderate**	Colocalization with molQTL of the target gene (H4 PP > 80%) Or Significant MR with genome-wide protein QTL (q-value < 0.05) Or Maximum ABC score for an element overlapping the lead variant Or Associated (*P* < 1 × 10^−6^) coding variant
**Weak**	Nearest gene to the lead variant Or Maximum ABC score for an element overlapping an associated variant (*P* < 1 × 10^−6^) Or ABC link (any score) between an element overlapping the lead variant and target gene
**Very weak**	Significant MR with eQTL Or ABC link (any score) between an element overlapping the lead variant and target gene

For a given locus, we then prioritized the best gene(s) as the one with the highest rank. In case of ties, we prioritized the nearest gene to lead variant if it is within the set of genes with highest scores, otherwise all highest ranked genes were prioritized equally.

### Enrichment of gold standard genes

We retrieved GWAS causal gene gold standards supported by functional experiments or observations or expert curation from Open Targets (version 191108) [26, 80]. We linked the current analysis with the gold standard gene list using Ensembl gene identifiers and EFO codes. That is, for a given gene-disease pair in the current analysis, we consider it a gold standard association if the gene and GWAS EFO code are present in the Open Targets gold standard gene-disease set. For each indication, we filtered out genes not represented in loci where a gold standard gene is located. We calculated the enrichment of gold standard genes in prioritized genes by different features or rankings as described above using Fisher exact tests. In addition, we calculated the precision (number of prioritized genes that are gold standards over all prioritized genes), recall (number of prioritized genes that are gold standards over the total number of gold standard genes), and F1 scores for each feature.

### Single gene colocalizing cell-type molQTL enrichment

To identify enriched cell types with colocalizing molQTL at single genes, we calculated the ratio of indications for which this gene is prioritized to be causal by a given molQTL dataset (H4 PP > 80%) over the total number of prioritized indications (as defined by unique EFO) for that gene. We collapsed GWAS by corresponding EFO code so that a gene was only counted once per indication (and not multiple times for GWAS of the same disease). We then compared this ratio to the fraction of prioritized indications via colocalization of the same eQTL dataset over all prioritized indications genome wide. In other words, we are looking for genes that show an overrepresentation of colocalizing eQTL cell types across all associated indications compared to the genome-wide distribution. This corresponds to the following contingency table:

$$\begin{array}{cc}\:{\sum\:}_{\mathit i}C_{\mathit i\mathit J\mathit K}&\:{\sum\:}_i{\sum\:}_{\mathit k\mathit\neq\mathit K}C_{\mathit i\mathit J\mathit K}\\\:{\sum\:}_i{\sum\:}_{\mathit j\mathit\neq\mathit J}C_{\mathit i\mathit j\mathit K}&\:{\sum\:}_i{\sum\:}_{k\mathit\neq K}{\sum\:}_{\mathit j\mathit\neq\mathit J}C_{\mathit i\mathit j\mathit k}\end{array}$$

Where *C*_*ijk*_=1 if disease *i* colocalize with prioritized gene *j* in tissue *k* and 0 if not. *P*-values and odds ratios were calculated using Fisher exact tests. False discovery rate (FDR) adjusted *P*-values < 0.05 were considered significant.

### Enrichment of disease categories for single genes

To identify enrichment of disease categories for single genes, we calculated the ratio of the number of GWAS where the genes is prioritized for a given EFO category over the total number of prioritized GWAS for that gene. We then compared this ratio to the genome-wide ratio of GWAS for this EFO category over the total number of tested GWAS. This corresponds to the following contingency table:

$$\begin{array}{cc}\:{\sum\:}_{\mathit i}D_{\mathit i\mathit J\mathit C}&\:\sum\nolimits_i{\sum\:}_{\mathit c\mathit\neq\mathit C}D_{\mathit i\mathit J\mathit c}\\\:{\sum\:}_i{\sum\:}_{\mathit j\mathit\neq\mathit\:\mathit J}D_{\mathit i\mathit j\mathit C}&\:{\sum\:}_{\mathit i}{\sum\:}_{c\mathit\neq C}{\sum\:}_{\mathit j\mathit\neq\mathit\:\mathit J}D_{\mathit i\mathit j\mathit c}\end{array}$$

Where *D*_*ijk*_=1 if disease *i* is prioritized for gene *j* and belongs to category *c* and 0 if not. *P*-values and odds ratios were calculated using Fisher exact tests. FDR adjusted *P*-values < 0.05 were considered significant.

### Disease colocalizing molQTL cell-type enrichment

We identify enriched cell types in GWAS disease EFO categories supported by colocalization as in King et al. 2021 [81]. Briefly, we extracted all GWAS colocalizing molQTL (H4 probability > 0.8). Then, for a given cell type *K* and disease category *I*, we generated the following contingency table:

$$\begin{array}{cc}\:{\sum\:}_j\;C_{IjK}&\:{\sum\:}_{j\;}{\sum\:}_{k\neq\:K}\;C_{Ijk}\\\:{\sum\:}_j{\sum\:}_{i\neq\:I}C_{ijK}&\:{\sum\:}_j{\sum\:}_{k\neq\:K}{\sum\:}_{i\neq\:I}C_{ijk}\end{array}$$

Where *C*_*ijk*_=1 if at least one disease GWAS of category *i* colocalize with gene *j* in tissue *k* and 0 if not. *P*-values and odds ratios were calculated using Fisher exact tests. We performed the analysis considering all molQTL separately, as well as by grouping similar cell types and tissues together prior to testing for enrichment. FDR adjusted *P*-values < 0.05 were considered significant.

### Drug target- indication pairs in clinical trials

Information about drugs approved or in clinical trials was obtained from the Citeline data from Informa Pharma Intelligence, which is a superset of the most used data sources. In addition to multiple data streams, including nightly feeds from official sources such as ClinicalTrials.gov, Citeline also contains data from primary sources such as institutional press releases, financial reports, study reports, and drug marketing label applications, and secondary sources such as analyst reports by consulting companies. Secondary sources are particularly important to reduce potential biases to the organizations’ tenancy to report only successful trials, especially those before the FDA Amendments Act of 2007, which requires all clinical trials to be registered and tracked by ClinicalTrials.gov. Citeline database contains information from both US and non-US sources. Any cancer or cancer related indications were excluded from this analysis.

In order to map gene-disease pairs in the genetic data to target-indication pairs in the drug data, we used EFO, which provided a systematic description of many data elements available in EBI databases. A target-indication pair is said to have genetic evidence if there is genetic evidence of association between the gene and disease sufficiently similar to the indication, based on semantic similarity. Two methods were used to calculate semantic similarity matrix [82, 83]. Semantic similarities between each pair of EFO headings were computed in the ontologySimilarity R package [84]. The average of the two methods was calculated and standardized similarities had a maximum value of 1 for each disease or indication. Two diseases are considered similar if the similarity is greater than or equal to a previously published value of 0.7 [11]. 

### Prediction of drug mechanism of action directionality

We retrieved information about drug mechanism of action (MoA) from the Informa Pharma Intelligence dataset described above. Drug MoA were linked to GWAS using a semantic similarity threshold 0.7. When multiple GWAS could be connected to the same drug target and indication, we kept only the GWAS with the most significant *p*-value at the locus. For targets for which *decreased* expression or loss of function (LoF) is beneficial, we considered datasets with the following keywords: “antagonist”, “inhibitor”, and “degrader”. For targets for which *increased* expression or function is beneficial, we considered the following keyworks: “agonist”, and “activator”. We considered drugs and targets in phase II clinical trial or above. We performed two analyses to infer directionality from GWAS. First, we assess directionality using the effect size of low-frequency lead coding variant (MAF < 5%). We assumed that these variants are disruptive or LoF. Therefore, a LoF coding variant associated with increased risk suggests that a drug MoA of agonist or activator would be beneficial, whereas for a protective LoF coding variant, an inhibitor or antagonist would be beneficial. Next, we assessed directionality based on the direction of effect of gene expression on disease risk predicted by MR using molQTL as exposure (q-value < 0.05). We included only molQTL colocalizing with local GWAS signal (H4 PP > 80%). For gene-disease pairs supported by multiple colocalizing molQTL, a consensus direction was inferred if the MR direction of effect was consistent across > 75% of the molQTL. Here, a negative consensus MR direction suggests that increased gene expression leads to decreased disease risk. Therefore, an activator or agonist drug targeting this gene would be beneficial. Conversely, a positive consensus MR direction suggests that increased gene expression increases disease risk, and an inhibitor or antagonist drug would be beneficial. We calculated enrichment of concordant direction of effect between GWAS and drug MoA using Fisher exact tests.

### Identification of causal links between diseases and genes related to the IL6 receptor

We aimed to apply our proposed approach to a specific case example. Using the causal gene prioritization and GWAS datasets described above, we extracted all disease GWAS for which *IL6*,* IL6R*, or *IL6ST* were predicted to be causal. We predicted directionality of effect of gene expression on disease risk by MR as above using a threshold of q-value < 0.05. We generated local association of plots molQTL and GWAS using LocusZoom [85]. We performed fine-mapping of *IL6ST* genetic variants associated with polymyalgia rheumatica using SuSIE [86] as previously described for FinnGen [7].

## Results

### Prioritization of putative causal genes in thousands of GWAS

**Fig. 1:**
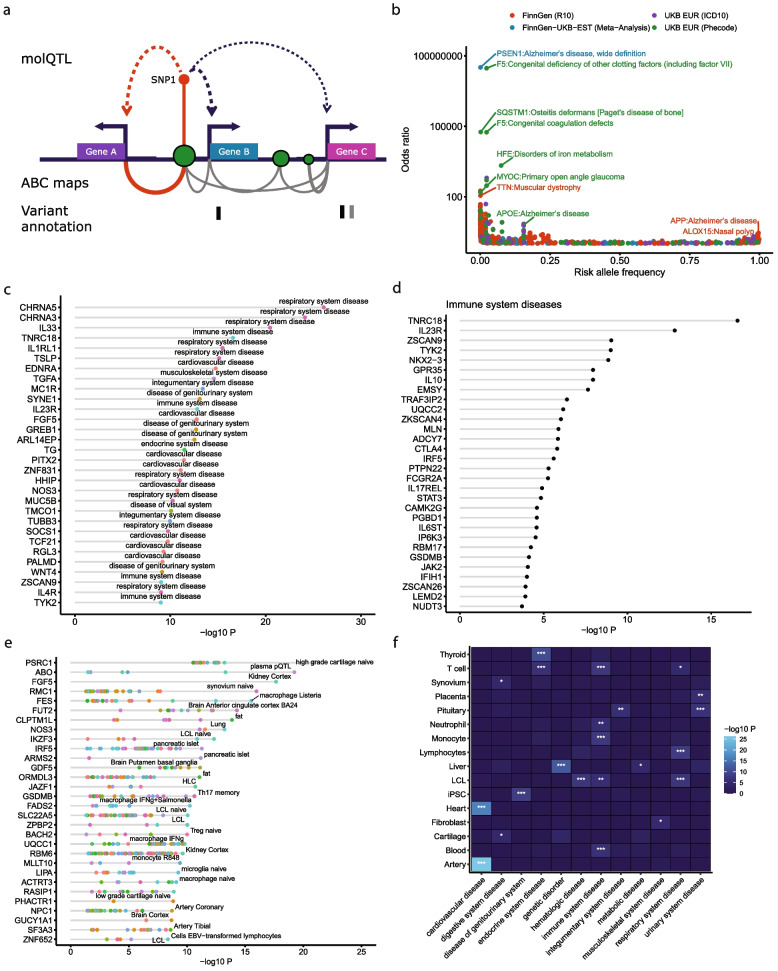
Characteristics of prioritized genes via gain or loss of function variants and molQTL.** A** Features used to prioritize genes in GWAS loci. Genes are ranked based on a combination of features including molQTL, activity-by-contact (ABC) maps, and variant annotations, including variant effect predictions (VEP) and pathogenicity predictions. **B** Disease-associated predicted pathogenic variants capture disease associations with high effect sizes. Lead pathogenic variants with GWAS *P*-value < 5 × 10^−8^ are reported in the figure. Effect of the risk allele (odds ratio) is reported on the y-axis. The x-axis corresponds to the frequency of the risk allele. **C** Disease category overrepresentation for single genes predicted to be causal. Each dot represents a different associated disease category. Top 30 enrichments are shown. **D** Same as B, but filtered for genes predicted to be causal and enriched in “Immune system diseases”. Each dot represents a different associated disease category. Top 30 genes are shown. **E** Overrepresentation of eQTL colocalization for single genes predicted to be causal. Gene-tissue pairs are included only if the gene has the highest rank in a locus for a given associated disease. Top 30 enriched eQTL are shown. Each dot represents a different enriched tissue or cell-type. **F** Enriched colocalizing cell types and tissues by disease categories. Only disease categories and tissues or cell types with at least one significant enrichment are reported in the heatmap. Enrichment *P*-values are calculated using Fisher exact test, testing for the enrichment of genes with eQTL colocalizing with GWAS belonging to specific disease categories as in [81]. Tissues and cell-types were collapsed into broader categories before testing for enrichment. For example, tibial, coronary, and aorta arteries were grouped into “artery” molQTL: Molecular QTL; ABC: Activity-By-Contact; LCL: Lymphoblastoid cell lines; iPSC: induced Pluripotent Stem Cells .: Adjusted *P* < 0.1; *: Adjusted *P* < 0.05; **: Adjusted *P* < 0.01; ***: Adjusted *P* < 0.001

We aimed to prioritize causal genes across 4,611 GWAS from 3 different sources (Table 1): UKB [45], FinnGen release 10 (R10), and meta-analyses of UKB, FinnGen R10, and Estonian biobank [6]. For simplicity, we refer to both single studies and meta-analyses as GWAS throughout the manuscript. While molQTL such as eQTL have been used previously to prioritize causal genes, they are often pleiotropic with the same variant associated with multiple genes within the same locus [26, 32, 33]. Additional genomic information such as the ABC model have been shown to increase performance to identify causal genes, in particular when selecting genes with the highest ABC score (ABCmax) [41]. Therefore, we derived a ranking scheme to prioritize genes using different features including ABC, molQTL, coding variant annotations and pathogenicity predictions, and distance to lead variant **(**Fig. 1A, methods). We integrated 110 molQTL datasets from 26 studies using MR to infer causality and directionality of gene expression on disease risk. We also performed colocalization analyses to confirm that both GWAS or meta-analyses and molQTL signals shared at least one causal variant. Top ranking genes were selected as those that either contained an associated lead coding variant or were supported by both ABCmax and colocalization across > 2 cell types or tissues. We did not include distance to lead variant for higher ranks because we wanted to first prioritize genes for which we could identify potential biological mechanisms. However, for loci without such evidence, or in cases where multiple genes showed identical ranks, the nearest gene to the lead variant was selected as the putative causal gene if it was among the best candidates. Overall, between 1.1 and 1.4 genes were prioritized per locus on average (before breaking ties with the nearest gene), with 17–45% of loci supported by molQTL colocalization or coding variants (Table 1).

**Table 1:** GWAS included in this study.The table reports the maximum GWAS sample size for each study, the total number of GWAS with at least one associated gene. The number of loci with at least one variant with GWAS *P* < 1 × 10^−6^. To calculate the number of loci, we defined 250 kb regions on each side of the lead variant. Overlapping regions were then merged. The table reports the total number of non-overlapping regions. The mean number of prioritized genes corresponds to the average number of genes prioritized across each GWAS. The mean number of prioritized gene per locus corresponds to the average number of genes with the highest scores in a locus. For the analyses reported throughout this manuscript, ties are broken using the shortest distance to the lead variant. Finally, the last column reports the average number of prioritized gene supported by coding variants or molQTL colocalization

Study ID	Max sample size	Number of GWAS	Mean *N *loci (*P* < 1 × 10^−6^)	Mean *N* prioritized genes	Mean *N* prioritized genes per locus	Mean *N* prioritized genes supported by molQTL or coding variants
FinnGen R10	412,181	2,297	16.36	19.9	1.17	0.21
FinnGen, UK biobank, Estonian biobank meta-analysis (R10)	1,073,998	95	123.44	164.76	1.32	0.44
UKBB pan ICD-10 (European)	420,531	898	9.01	10.23	1.08	0.17
UKBB pan phecodes (European)	42,0531	1,321	10.52	12.21	1.09	0.19

*molQTL *molecular QTL, *N *Number

### Enrichment of genomic features for gold standard genes

Comparing the enrichment of different genomic features alone for curated gold standard genes [26], we found a strong enrichment for genes supported by ABCmax with lead variant (Odds ratio (OR) = 16.3, *P* = 5 × 10^−19^i (Additional file 1: Figure S1; Additional file 2: Table S1). molQTL colocalization also enriched for gold standard genes (colocalization H4 posterior probability (PP) > 95%, OR = 13.3, *P* = 3 × 10^−31^). However, the strongest enrichment was observed for genes with associated coding variants (OR = 50.5, *P* = 7 × 10^−60^) and the nearest genes (OR = 28.5, *P* = 3 × 10^−81^). These strong enrichments are expected given that the gene closest to the lead variant is often the causal gene. In addition, several of the gold standard genes have been selected because they are supported by coding variants or tend to fall in the center of GWAS peaks and have been investigated more closely [26]. However, when using these features in combination, we found that our ranking approach performed well and generally better than selecting the nearest gene alone, with a mean increase in F1 score of 0.13 across studies (range 0.08–0.23) (Additional file 1: Figure S2-S3; Additional file 2: Table S1).

### Pathogenicity annotations identify genes linked to monogenic disorders

Integrating information about variant pathogenicity retrieved variants linked to monogenic disorders including *PSEN1* with Alzheimer’s disease (AD) [87] (rs764971634, p.Ile437Val, *P* = 2 × 10^−12^), *SQSTM1* and Paget’s disease [88] (rs104893941, p.Pro392Leu, *P* = 6 × 10^−11^ ), and *HFE* and disorders of iron metabolism [89] (rs1800562, p.Cys282Tyr, *P* = 1 × 10^−178^) (Fig. 1B; Additional file 2: Table S3). We also identified protective variants such as *APP* p.Ala673Thr (rs63750847, *P* = 7 × 10^−11^) reducing odds of developing AD [90], and *ALOX15* p.Thr560Met protecting against nasal polyps (rs34210653, *P* = 2 × 10^−15^) [91]. Of 504 genes prioritized with at least one predicted pathogenic lead variant, 287 had at least one disease mutation reported in the Human Gene Mutation Database (HGMD) [50] (OR = 2.4 [2.0-2.9], *P* = 4 × 10^−21^). Potential novel associations included *COLGALT2* and arthrosis (rs35937944, p.Tyr212Cys, *P* = 2 × 10^−14^), *LGR5* and carcinoid syndrome (rs200138614, p.Cys712Phe, *P* = 4 × 10^−9^), and *GREB1* and female infertility (rs755857714, p.Arg1339His, *P* = 4 × 10^−9^).

### Colocalizing molQTL link genes to diseases and pathogenic tissues

Prioritized candidate causal genes showed enrichment in disease-colocalizing molQTL related to their known function. For instance, colocalizing molQTL for prioritized genes supported associations with disease categories such as *EDNRA*, *LPA* and *FGF5* with cardiovascular diseases (*P* < 5 × 10^−10^), *TSLP*, *IL33*, *CHRNA3*, and *CHRNA5* and respiratory system diseases (*P* < 2 × 10^−16^), and *IL23R*,* TYK2*,* IL10* and immune system disease (*P* < 2 × 10^−9^) (Fig. 1C-D; Additional file 2: Table S4**)**. In addition, disease-colocalizing molQTL tended to be enriched in specific tissues and cell types. For instance, we found an enrichment of disease-colocalizing eQTL in kidney cortex for *FGF5*, a gene expressed during kidney development and associated with kidney function (*P* = 2 × 10^−18^) [92] (Fig. 1E; Additional file 2: Table S5). Other examples include artery eQTL for the cardiovascular diseases associated gene *PHACTR1* [93] (*P* = 8 × 10^−10^); the lysosomal acid lipase (*LIPA*) gene and microglia eQTL (*P* = 2 × 10^−11^); and the *ABO* blood group gene with plasma pQTL (*P* = 1 × 10^−21^). Finally, we confirmed that enriched colocalizing eQTL matched the expected pathogenic tissues and cell-types of different disease categories (Fig. 1F; Additional file 2: Table S6). For instance, after grouping eQTL of similar tissues and cell types together, we found a strong enrichment of genes with artery and heart eQTL colocalizing with cardiovascular disease GWAS (*P* < 6 < x10^−17^). We found similar enrichment for T cell and thyroid eQTL in endocrine system diseases (*P* < 3 × 10^−7^); blood, lymphoblastoid cell line, monocytes, neutrophil, and T cells with immune system diseases (*P* < 1 × 10^−6^); and fibroblasts and musculoskeletal diseases (*P* = 7 × 10^−6^). Treating each eQTL dataset separately revealed additional associations with tissues or cell subsets including brain cortex and diseases of the visual system (*P* = 7 × 10^−6^); cerebellum and nervous system diseases (*P* = 4 × 10^−6^); regulatory T cells and endocrine system diseases (*P* = 9 × 10^−9^); and T helper 17 cells and digestive system diseases (*P* = 5 × 10^−7^) (Additional file 1: Figure S4; Additional file 2: Table S7). Overall, the analyses illustrate that in contrast to the nearest gene approach, inclusion of molQTL can help contextualize genetic associations to potential pathogenic cell types and tissues.

### Prioritized genes increase clinical trial probability of success

**Fig. 2:**
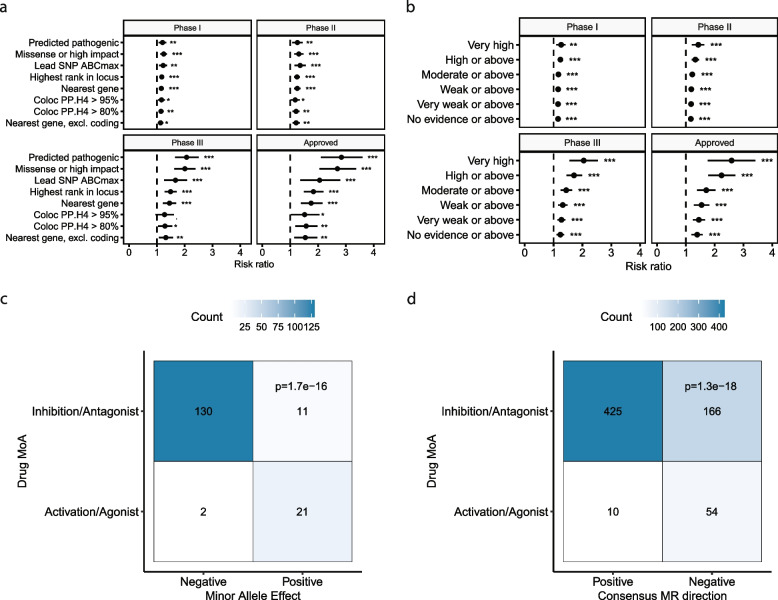
Prioritized genes predict clinical trial success.** A** Enrichment of targets of approved drugs or drugs in clinical trials (phase I-III) using genetic evidence aggregated from FinnGen, UKB, and biobank meta-analyses prioritizing genes using colocalization (posterior probability of colocalization [H4] > 80% or > 95%), predicted pathogenic variants, genes with highest prioritization rank, ABC score for lead variant, or nearest gene excluding loci with associated coding variants. **B** Enrichment of targets of approved drugs or drugs in clinical trials (phase I-III) using causal gene prioritization ranks across all studies. **C** Concordance between direction of effect of lead low-frequency coding variants on disease risk, and drug MoA for targets in phase II clinical trials or above. We retrieved information about targets, clinical trials, and drug MoA from the Citeline Pharmacogenomics dataset. We connected this dataset to GWAS phenotypes using EFO codes and a semantic similarity score > 0.7. We assume that low-frequency coding variants (minor allele frequency < 5%) are disruptive (LoF). Therefore, a negative (protective) direction of effect would translate into inhibition or antagonism being beneficial (and vice-versa). **D** Concordance between the predicted impact of gene expression on disease risk predicted by MR, and drug MoA for targets in phase II clinical trials or above. Information about targets, clinical trials, and drug MoA were collected from the Citeline Pharmacogenomics dataset and connected to GWAS phenotypes using EFO codes and a semantic similarity score > 0.7. The direction of effect of gene expression on disease risk was assessed by MR using molQTL as exposure (q-value < 0.05). Only molQTL colocalizing with local GWAS signal (H4 posterior probability > 80%) were included. A consensus direction was inferred if the MR direction of effect was consistent across > 75% of molQTL for a given gene and disease GWAS. A negative consensus MR direction suggests that increased gene expression leads to decreased disease risk. Therefore, an activator or agonist drug targeting this gene would be beneficial. Conversely, a positive consensus MR direction suggests that increased gene expression increases disease risk, and an inhibitor or antagonist drug would be beneficial. Reported *P*-values were calculated by Fisher exact test .: *P* < 0.1; *: *P* < 0.05; **: *P* < 0.01; ***: *P* < 0.001

Building on these results, we tested whether we could use molQTL information of putative causal gene to drive drug repurposing opportunities. First, we evaluated whether the prioritized genes enriched for therapeutic targets with clinical trial success. Clinical trial information was retrieved from the Citeline Pharma Intelligence project. Consistent with previous observations, we found that targets with clinical trial success were enriched for features such as presence of coding variants (Fig. 2A, Additional file 2: Table S8). For example, likely pathogenic coding variants demonstrated some of the best predictive performances (Phase I: Risk ratio (RR) = 1.20, *P* = 0.007; Phase II: RR = 1.26, *P* = 0.008; Phase III: RR = 2.07, *P* = 3 × 10^−8^; Approved: RR = 2.84, *P* = 1 × 10^−9^). Similar results were observed analyzing each study separately (Additional file 1: Figure S5). Use of epigenetic evidence also improved predictions, in particular lead SNPs linked by the ABC model (Phase I: RR = 1.23, *P* = 0.004; Phase II: RR = 1.36, *P* = 6 × 10^−4^; Phase III: RR = 1.67, *P* = 5 × 10^−4^; Approved: RR = 2.06, *P* = 4 × 10^−4^). However, molQTL information alone did not enrich as much for clinical trial success, for example, colocalizing molQTL with posterior probability > 80% (Phase I: RR = 1.15, *P* = 0.003; Phase II: RR = 1.21, *P* = 0.001; Phase III: RR = 1.29, *P* = 0.01; Approved: RR = 1.57, *P* = 0.002). While the overall prioritized genes did not show the strongest enrichment (Phase I: RR = 1.18, *P* = 1 × 10^−5^; Phase II: RR = 1.24, *P* = 3 × 10^−6^; Phase III: RR = 1.49, *P* = 7 × 10^−7^; Approved: RR = 1.83, *P* = 4 × 10^−8^), this was likely due to the inclusion of genes with no supportive evidence other than distance (Fig. 2A). Indeed, we found that “High” and “Very High” prioritization ranks were more predictive of successful clinical trial progression (higher risk ratios) than lower-ranking genes, especially at later clinical trial phases or after approval (High + Very high ranks Phase I: RR = 1.24, *P* = 4 × 10^−6^; Phase II: RR = 1.33, *P* = 8 × 10^−7^; Phase III: RR = 1.71, *P* = 3 × 10^−8^; Approved: RR = 2.24, *P* = 1 × 10^−10^) (Fig. 2B, Additional file 1: Figure S5, Additional file 2: Table S9). In our analysis, distance itself was not as predictive of clinical trial success especially after excluding loci likely driven by coding variants (Phase I: RR = 1.13, *P* = 0.01; Phase II: RR = 1.21, *P* = 0.001; Phase III: RR = 1.32, *P* = 0.009; Approved: RR = 1.54, *P* = 0.003) (Fig. 2B).

### Inferred directionality from GWAS recapitulate drug MoA

To understand whether inferred directionality could be informative of clinical trial success, we first investigated the consistency between the direction of effect of coding variants and drug MoA (methods). When considering prioritized genes with lead low-frequency coding variants (minor allele frequency < 0.05) and clinical trials phase II and above, between 92% showed consistent effect between the minor allele and drug MoA (Fisher *P* = 2 × 10^−16^, Fig. 2C). Results were similar when stratifying GWAS by data source (Additional file 1: Figure S6). We then asked whether molQTL could similarly inform on directionality. Using prioritized gene-disease pairs supported by MR (q-value < 0.05) and colocalization (PP > 80%), we inferred the direction of effect when the predicted MR effect was consistent across > 75% of molQTL datasets for a given gene. This was the case for most gene-disease pairs (Additional file 1: Figure S7). Again, direction of effect was generally in agreement with drug MoA (73% agreement, Fisher *P* = 4 × 10^−8^-5 × 10^−41^, Fig. 2D, Additional file 1: Figure S6). Consistency across all studies increased when considering only approved drugs (85–94% agreement, Fisher *P* = 4 × 10^−7^-5 × 10^−26^, Additional file 1: Figure S8). Overall, these data suggest that molQTL can be used to inform on drug MoA.

### Causal gene predication from GWAS identifies a link between IL6ST and polymyalgia rheumatica

**Fig. 3:**
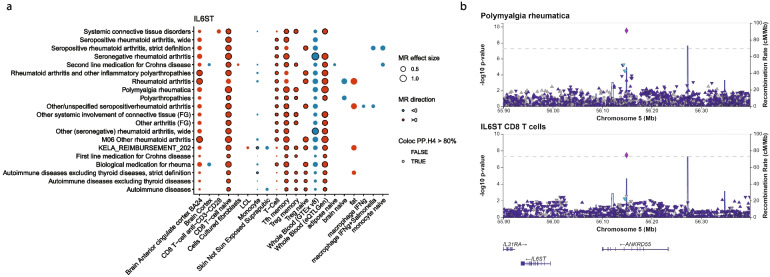
*IL6ST *is predicted to be causal for rheumatoid arthritis and polymyalgia rheumatica.** A** Diseases associations supported by MR, colocalization and ABC. The figure shows tissues and cell-types with significant MR (q-value < 0.05) using *IL6ST* eQTL as exposure and diseases as outcome (red: positive effect size estimate [MR beta]; blue: negative effect size estimate). The size of the dots represents absolute effect size. Disease-eQTL pairs with a colocalization posterior probability > 80% are highlighted with a dark border. **B** LocusZoom [85] plot showing the top association for polymyalgia rheumatica at the *ANKRD55*-*IL6ST* locus. Both *IL6ST* and *ANKRD55* eQTL colocalize with the polymyalgia rheumatica signal, but *IL6ST* has the highest ABC score

Finally, we applied our causal gene prioritization approach to a specific use case, that is to identify potential new indications for drugs targeting the IL6 receptor such as Sarilumab and Tocilizumab, both drugs approved for rheumatoid arthritis. We extracted diseases prioritized by our approach for genes related to the receptor, namely *IL6*, *IL6ST*, and *IL6R*. We identified putative causal links between increased *IL6* expression in CD16 monocytes and increased risk of varicose veins, ischemic heart disease, coronary atherosclerosis, and atrial fibrillation (MR beta > 0), but decreased risk of asthma and allergy (MR beta < 0) (Additional file 1: Figure S9; Additional file 2: Table S10). eQTL of *IL6* in whole blood also supported these disease associations, albeit with an opposite predicted direction of effect. Similarly, *IL6R* expression in multiple tissues including artery, colon, and esophagus was associated with increased risk of coronary revascularization, coronary atherosclerosis, and abdominal aortic aneurysm (AAA), but lower risk of lower respiratory diseases and atopic dermatitis (Additional file 1: Figure S10). Again, we observed opposite direction of effect predicted by MR for these diseases when using monocyte or macrophage eQTL as exposure. The associations with coronary atherosclerosis and AAA were further driven by a lead coding variant in *IL6R*, rs2228145 (Asp358Ala, Additional file 2: Table S10). Finally, we found that increased *IL6ST* expression in T cells and whole blood is predicted to increase the risk of rheumatoid arthritis, systemic connective tissue disorders, polyarthropathies, other arthritis, autoimmune diseases, and polymyalgia rheumatica (Fig. 3A, Additional file 2: Table S10). These associations were driven by rs7731626 (SuSIE fine-mapping probability > 0.99). This variant is located within an intron of *ANKRD55* and colocalizes with eQTL for both *ANKRD55* and *IL6ST* (PP > 80%). However, this variant also overlaps with an enhancer that shows the highest ABC score for *IL6ST* for genes in the region, suggesting the latter is the causal gene, in line with previous studies [94, 95] (Fig. 3B). Overall, our approach was able to capture known associations with IL6-R related genes and identified an association between *IL6ST* and polymyalgia rheumatica.

## Discussion

We prioritized disease-associated genes across 4,611 GWAS and meta-analyses from biobank studies using a combination of MR with molQTL, colocalization analysis, variant effect prediction, and epigenetic annotations (ABC model). This approach allows the use of molQTL to infer directionality of gene expression on disease risk, while improving the causal gene prediction compared to using molQTL alone. Based on combination of these features, we used a ranking approach to prioritize genes within loci and showed that this approach is enriched for gold standard genes. We recover known coding variant associations, including rare variants in genes linked to monogenic disorders such as *PSEN1* and *APP1* and Alzheimer’s disease, and *SQSTIM1* and Paget’s disease (Fig. 1B). Genes prioritized by molQTL also show enrichment in disease categories related to their function with pathogenic tissue contexts (Fig. 1C-F). Of note, when multiple genes show evidence of colocalization within the same locus, the addition of epigenetic (ABCmax) information can help prioritize one gene over the others. We note as an example the association of variants with polymyalgia rheumatica at the *ANRKD55* locus where this gene would be prioritized using the nearest gene approach. Whereas colocalization alone did not identify a single causal gene, combination of colocalization and ABCmax identified *IL6ST* as the putative causal gene, consistent with recent reports [96, 97]. *IL6ST* encodes a protein involved in signal transduction for the IL6 receptor pathway. Inhibitors of the IL6 receptor have recently shown success in clinical trials for this indication leading to a recent approval by the FDA [98]. 

In line with previous studies [11, 12], we show that therapeutic targets with genetic evidence are enriched at later clinical trial phases and as targets of approved drugs. In our analysis, using the nearest gene information alone was not strongly predictive of clinical trial success. The most predictive features were coding variant annotations and ABC maps. While the latter performs well to link causal genes to diseases, it does not provide information about directionality. We used coding variants and MR with molQTL to infer directionality of a target on disease risk. Both approaches were generally consistent with drug MoA matched for the target and disease. These data support that molQTL can be used to predict drug MoA. However, while we found that in general eQTL were consistent across cell type and tissues for a given gene and disease (Additional file 1: Figure S7), we note that this isn’t always the case. This is exemplified by the IL6-R case study, where all three queried genes displayed inconsistent direction of effect predicted by MR depending on the molQTL dataset. Future improvement of this approach should consider prior knowledge on pathogenic cell types or tissues to infer directionality in relevant contexts. Overall, our analysis suggests that using features such as ABCmax in combination with molQTL can increase the performance of causal gene inference approaches while informing on directionality which is crucial for translating GWAS hits to therapies.

We note that this study has some limitations. First, we did not perform fine-mapping analyses nor colocalization approaches that use LD references. Indeed, we opted to avoid methods that do not rely on LD references as we used GWAS from various sources, including meta-analyses where these methods may not be well calibrated [99]. Nevertheless, using fine-mapping information likely would improve performance, especially in cases where there are multiple causal variants underlying molQTL or GWAS signals, and would reduce LD contamination [30, 100]. In addition, using MR approaches like SMR and HEIDI or MRLocus, are likely to perform better in case of pleiotropy or allelic heterogeneity [101, 102]. This is evident in the case of *IL6ST*, where MR using eQTL from whole blood from different sources (GTEx, eQTLGen) lead to inversed estimate of directionality (Fig. 3A). This difference was due to different instruments used as only one genetic instrument was included in GTEx whereas 5 independent instruments were included for eQTLGen. We also assume that there is one causal gene per locus, although it is possible that multiple genes contribute to disease risk. Finally, integrating other sources of molQTL such as metabolite or splice QTL could help further identify putative causal genes as coding variants and eQTL only cover a fraction of loci (17–47% in this study) [103]. Similarly, considering additional cell types in both the molQTL and ABC annotations would further help identify functional links between variants and genes. While these approaches can be useful to nominate candidate causal genes and their relationship to diseases, proper functional validation remains of high importance.

## Conclusions

We nominated putative causal genes across 4,611 GWAS from biobank studies and public resources by integrating variant annotations as well as molQTL. We show that these prioritized genes recover known biological relationships in terms of disease and tissue enrichment and are enriched for therapeutic targets that succeeded in clinical trials. We show that directionality predicted by molQTL and coding variants generally recapitulate drug MoA. Finally, we applied this approach to genes related to the IL6 receptor and identified an association between *IL6ST* and polymyalgia rheumatica supporting the recent approval of Sarilumab for this indication.

## Supplementary Information

Additional file 1: Figure S1. Gold standard gene enrichment by genomic features. Figure S2. Precision and recall of gold standard genes for different genomic features as well as causal candidate prioritization approach. Figure S3. F1 scores for each considered features and prioritization scheme. Figure S4. Enriched colocalizing cell types and tissues by disease categories. Figure S5. Enrichment of clinical success stratified by GWAS source. Figure S6. Predicted directionality and drug mechanism of action stratified by GWAS source. Figure S7. Predicted direction of effect of gene expression on disease risk. Figure S8. Concordance between the predicted effect of gene expression on disease risk by MR and mMoA of approved drugs. Figure S9. Association between *IL6* and diseases, supported by MR, colocalization and ABC. Figure S10. Association between *IL6R* and diseases, supported by MR, colocalization and ABC.

Additional file 2: Table S1. Enrichment of gold standard genes by feature and GWAS study source. Table S2. Precision and recall of different features to recover gold standard genes. Table S3. Genes with predicted gain or loss of function variants (*P*<1x10^-6^). Table S4. Genes with overrepresented disease categories of GWAS in which they are prioritized as causal. Table S5. Genes with overrepresented cell-type colocalizing QTL with GWAS in which they are prioritized as causal. Table S6. Significantly enriched colocalizing QTL cell types and tissues in disease GWAS categories, after grouping similar tissues and cell-types together. Table S7. Significantly enriched colocalizing QTL cell types and tissues in disease GWAS categories, treating each eQTL dataset separately. Table S8. Enrichment of prioritized genes by feature across clinical trial phases and approved drugs. Table S9. Enrichment of prioritized genes by rank across clinical trial phases and approved drugs. Table S10. Putative causal association between diseases and IL6, IL6ST, or IL6R.

Additional file 3. List of FinnGen authors and their affiliations.

Additional file 4. Funding statements and references for all eQTL and pQTL datasets used for this manuscript.

## Data Availability

The UK Biobank Pan ancestry GWAS are available through https://pan.ukbb.broadinstitute.org/. FinnGen GWAS are available through https://www.finngen.fi/en/access_results. Processed and formatted eQTL data used in this study are available through the eQTL catalogue https://www.ebi.ac.uk/eqtl/. pQTL from Sun et al. 2018 are available through http://www.phpc.cam.ac.uk/ceu/proteins/. eQTLGen eQTL are available through https://www.eqtlgen.org/phase1.html. 1000 Genomes project phase 3 data [79] is available through https://www.internationalgenome.org/category/phase-3/. Activity-by-contact maps are available through https://www.engreitzlab.org/resources/. ProtVar annotations, including ESMb1 and EVE, are available through https://www.ebi.ac.uk/ProtVar/. Code for GWAS gene prioritization is available at https://github.com/Sanofi-Public/PMCB-GWAS_multi-omics_prioritization. Datasets supporting the conclusions of this article are included within the article and its additional files. Additional datasets used and/or analyzed during the current study are available from the corresponding author on reasonable request.

## Abbreviations

AAA: Abdominal aortic aneurysm
ABC: Activity-by-contact
CI: Confidence interval
EFO: Experimental factor ontology
eQTL: Expression quantitative trait loci
EstBB: Estonian Biobank
GWAS: Genome-wide association study
GoF: Gain of function
HLA: Human leukocyte antigen
iPSC: Induced Pluripotent Stem Cells
LCL: Lymphoblastoid cell lines
LD: Linkage disequilibrium
LoF: Loss of function
MAF: Minor allele frequency
MoA: Mechanism of action
MR: Mendelian randomization
molQTL: Molecular quantitative trait loci
OR: Odds ratio
pQTL: Protein quantitative trait loci
PP: Posterior probability
QTL: Quantitative trait loci
RR: Risk ratio
UKB: UK Biobank
VEP: Variant effect predictor
