# Supplementary material for: Leveraging large-scale multi-omics evidences to identify therapeutic targets from genome-wide association studies
Source: BMC Genomics. 2024 Nov 19;25:1111. doi: 10.1186/s12864-024-10971-2 (PMC11577829; doi:10.1186/s12864-024-10971-2)
Supplement: Supplementary file 1 — Additional file 1: Figure S1. Gold standard gene enrichment by genomic features. Figure S2. Precision and recall of gold standard genes for different genomic features as well as causal candidate prioritization approach. Figure S3. F1 scores for each considered features and prioritization scheme. Figure S4. Enriched colocalizing cell types and tissues by disease categories. Figure S5. Enrichment of clinical success stratified by GWAS source. Figure S6. Predicted directionality and drug mechanism of action stratified by GWAS source. Figure S7. Predicted direction of effect of gene expression on disease risk. Figure S8. Concordance between the predicted effect of gene expression on disease risk by MR and mMoA of approved drugs. Figure S9. Association between IL6 and diseases, supported by MR, colocalization and ABC. Figure S10. Association between IL6R and diseases, supported by MR, colocalization and ABC. [file 12864_2024_10971_MOESM1_ESM.docx]

**SUPPLEMENTAL FIGURES**


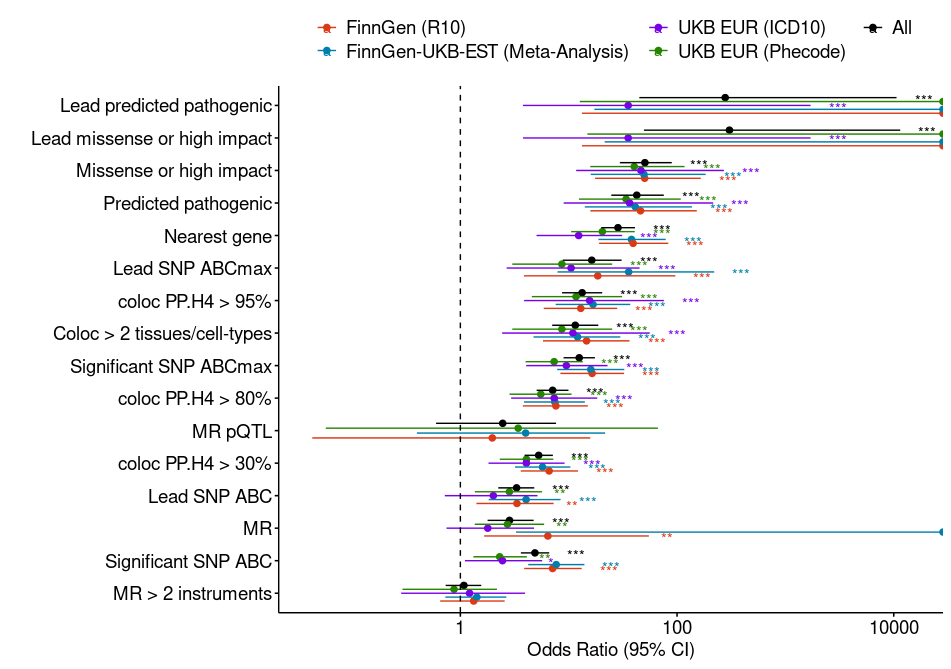
**Figure S1. Gold standard gene enrichment by genomic features.** The figure shows the odds ratio (OR) of recovering gold standard genes[1] for each feature separately. For predicted pathogenic variants and in some cases of lead missense or high impact variant and MR, features recovered all gold standard genes, leading to infinite oOR. Predicted pathogenic variants were defined as those with high impact based on Variant Effect Predictor[3] or missense variant annotations from ProtVar[48], considering conservation, structure stability predictions, and EVE[49] and ESM1b scores[50]. Nearest genes correspond to gene with their transcription start site nearest to the GWAS lead variant. Coloc > 2 tissues or cell types indicates loci with a colocalization posterior probability (H4) > 80% using eQTL from >2 different cell types or tissues.

Coloc: Colocalization; MR mendelian randomization; ABC: Activity-by-contact; CI: confidence interval.; PP.H4: Posterior probability of colocalization; . : P<0.1; *: P<0.05; **: P<0.01; ***: P<0.001

**
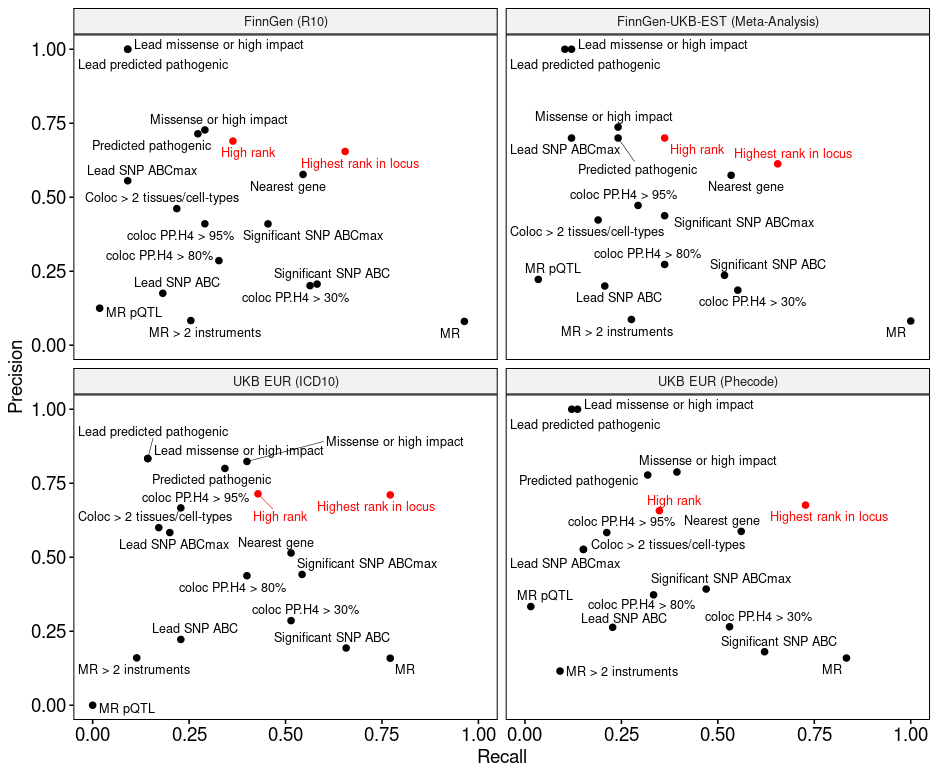
**

**Figure S2. Precision and recall of gold standard genes for different genomic features as well as causal candidate prioritization approach**. High rank references to genes prioritized for a given disease GWAS locus supported by colocalization >2 cell types and ABC interactions or coding variants (“high” or “very high” ranks, see METHODS). Highest rank in locus refers to genes prioritized for a given disease GWAS locus regardless of rank.

Coloc: Colocalization; MR mendelian randomization; ABC: Activity-by-contact; PP.H4: Posterior probability of colocalization.


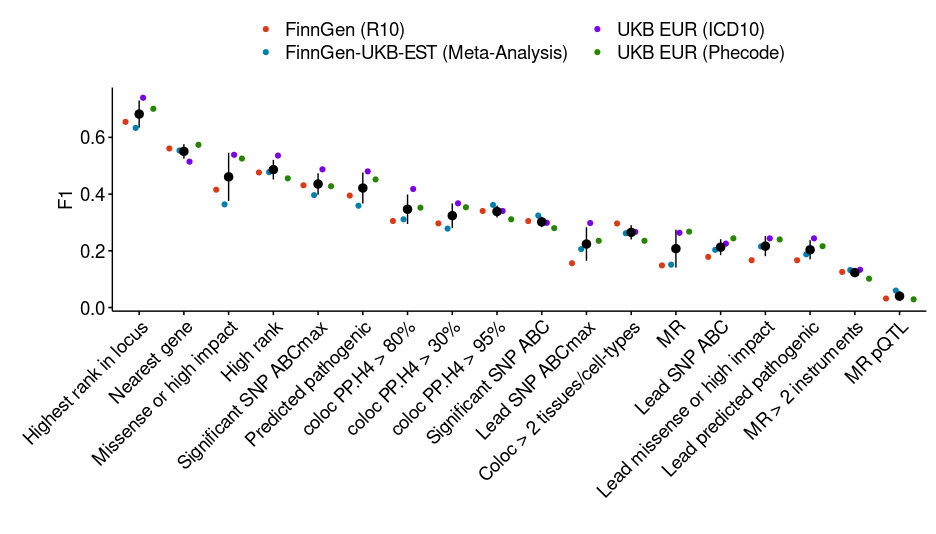
**Figure S3. F1 scores for each considered features and prioritization scheme.** “Highest rank in locus” corresponds to the best scoring gene(s) for a given GWAS within a particular locus. “High rank” corresponds to genes with “high” or “very high” ranks, that is genes supported by an associated coding variant or both ABC and colocalization > 2 cell types or tissues.

Coloc: Colocalization; MR mendelian randomization; ABC: Activity-by-contact; AUC: Area under the curve.


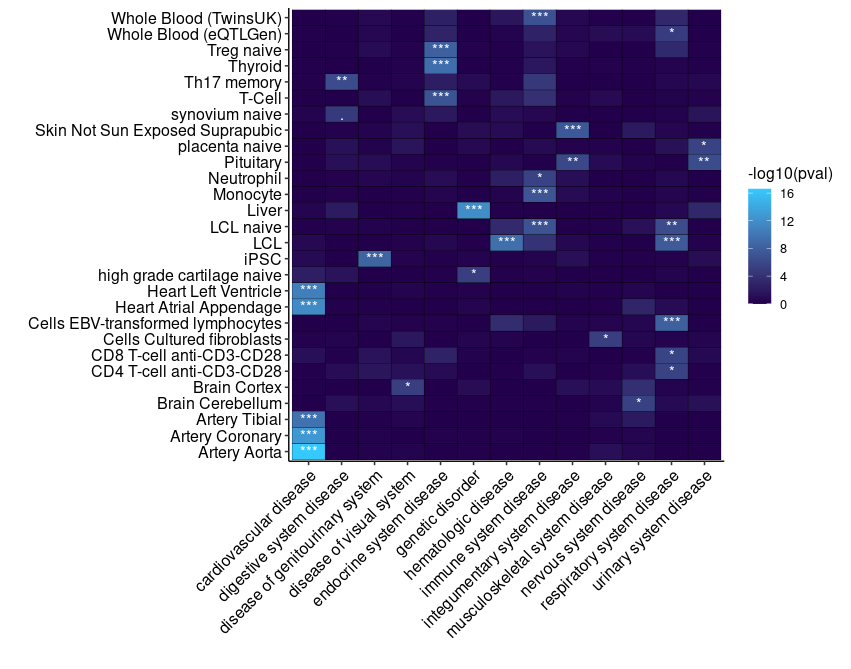


**Figure S4**. **Enriched colocalizing cell types and tissues by disease categories.** Only disease categories and tissues or cell types with at least one significant enrichment are reported in the heatmap. Enrichment *P*-values are calculated using Fisher exact test, testing for the enrichment of genes with eQTL colocalizing with GWAS belonging to specific disease categories as in [4].

Adjusted P<0.1; *: Adjusted P<0.05; ** Adjusted P<0.01; *** Adjusted P<0.001


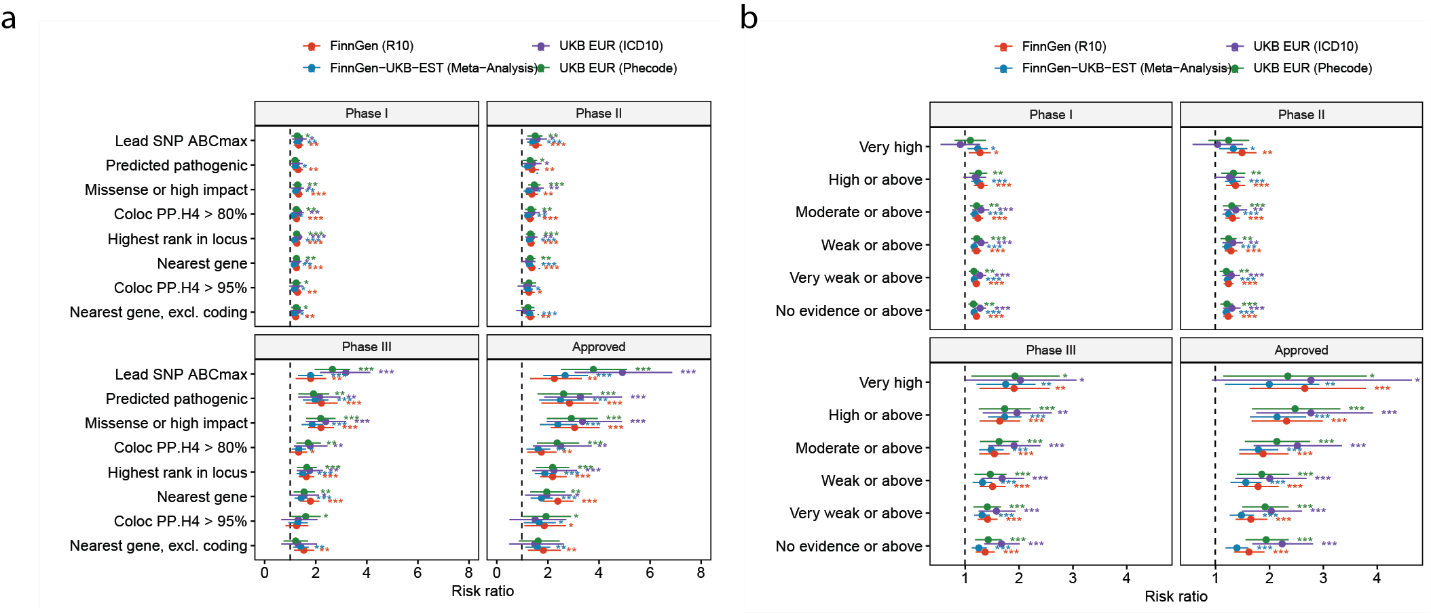


**Figure S5**. **Enrichment of clinical success stratified by GWAS source. a)** Enrichment of targets of approved drugs or drugs in clinical trials (phase I-III) using genetic evidence from FinnGen, UKB, and biobank meta-analyses prioritizing genes using colocalization (posterior probability of colocalization [H4] > 80% or > 95%), predicted pathogenic variants, genes with highest prioritization rank, ABC score for lead variant, or nearest gene excluding loci with associated coding variants. **B)** Enrichment of targets of approved drugs or drugs in clinical trials (phase I-III) using causal gene prioritization ranks in FinnGen, UKB, and biobank meta-analyses.

.: P<0.1; *: P<0.05; **: P<0.01; ***: P<0.001


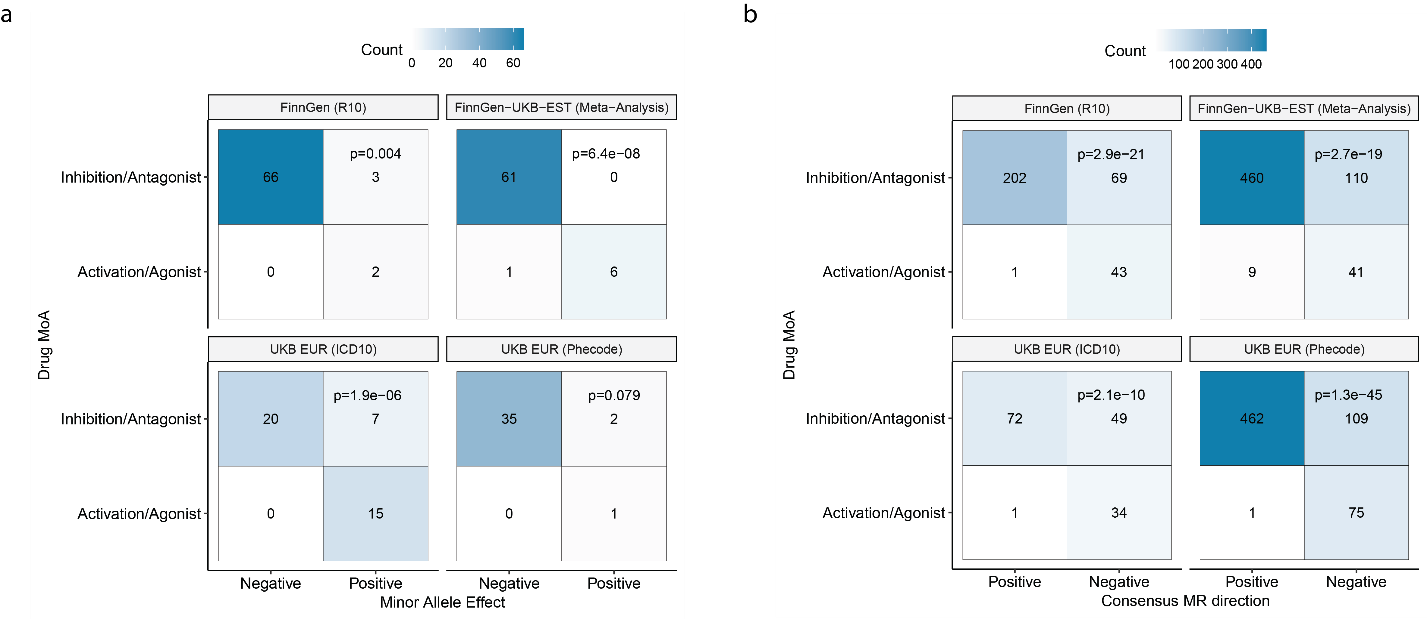


**Figure S6**. **Predicted directionality and drug mechanism of action stratified by GWAS source. a)** Concordance between direction of effect of lead low-frequency coding variants on disease risk, and drug MoA for targets in phase II clinical trials or above. **b)** Concordance between the predicted impact of gene expression on disease risk predicted by MR, and drug MoA for targets in phase II clinical trials or above. Reported *P*-values were calculated by Fisher exact test.

**
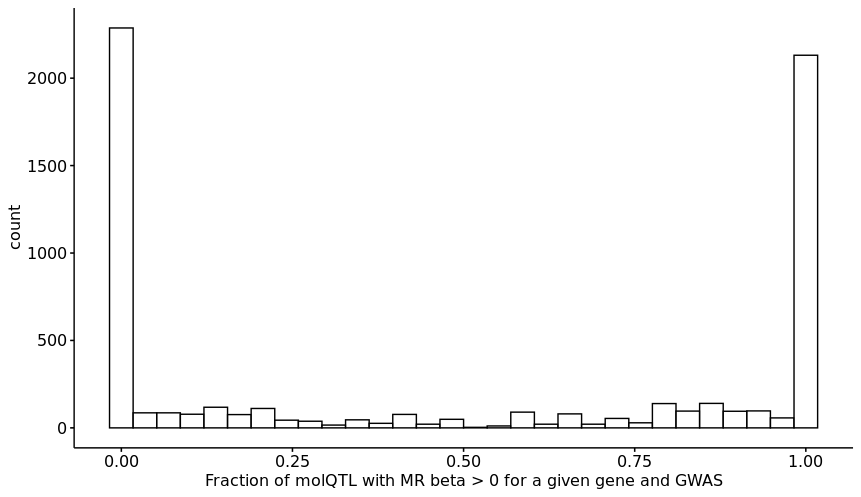
**

**Figure S7. Predicted direction of effect of gene expression on disease risk.** For a given gene and disease GWAS, we used mendelian randomization with molQTL (expression or protein QTL) as exposure to infer the impact of expression on disease risk (q-value < 0.05). We only included molQTL which colocalized with the local GWAS signal with a H4 posterior probability > 80%. For each gene and disease, we then calculated to fraction of colocalizing molQTL that were predicted to increase disease risk i.e. a fraction of 0 means that increased expression of gene X is predicted to decrease risk of disease Y across all molQTL datasets assessed. Conversely, a fraction of 1 means that increased expression of gene X is predicted to increase risk of disease Y across all molQTL assessed. We only include gene-GWAS pairs for which there were at least 5 colocalizing molQTL.

molQTL: Molecular quantitative trait loci; MR : Mendelian randomization; GWAS : Genome-wide association study

**
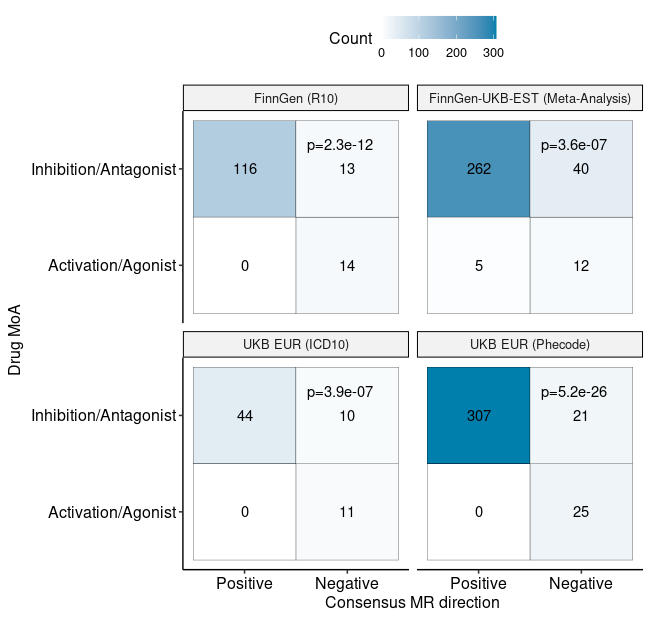
**

**Figure S8. Concordance between the predicted effect of gene expression on disease risk by MR and MoA of approved drugs.** Reported P-values were calculated by Fisher exact test.

MR: Mendelian randomization; MoA: Mechanism of action

**
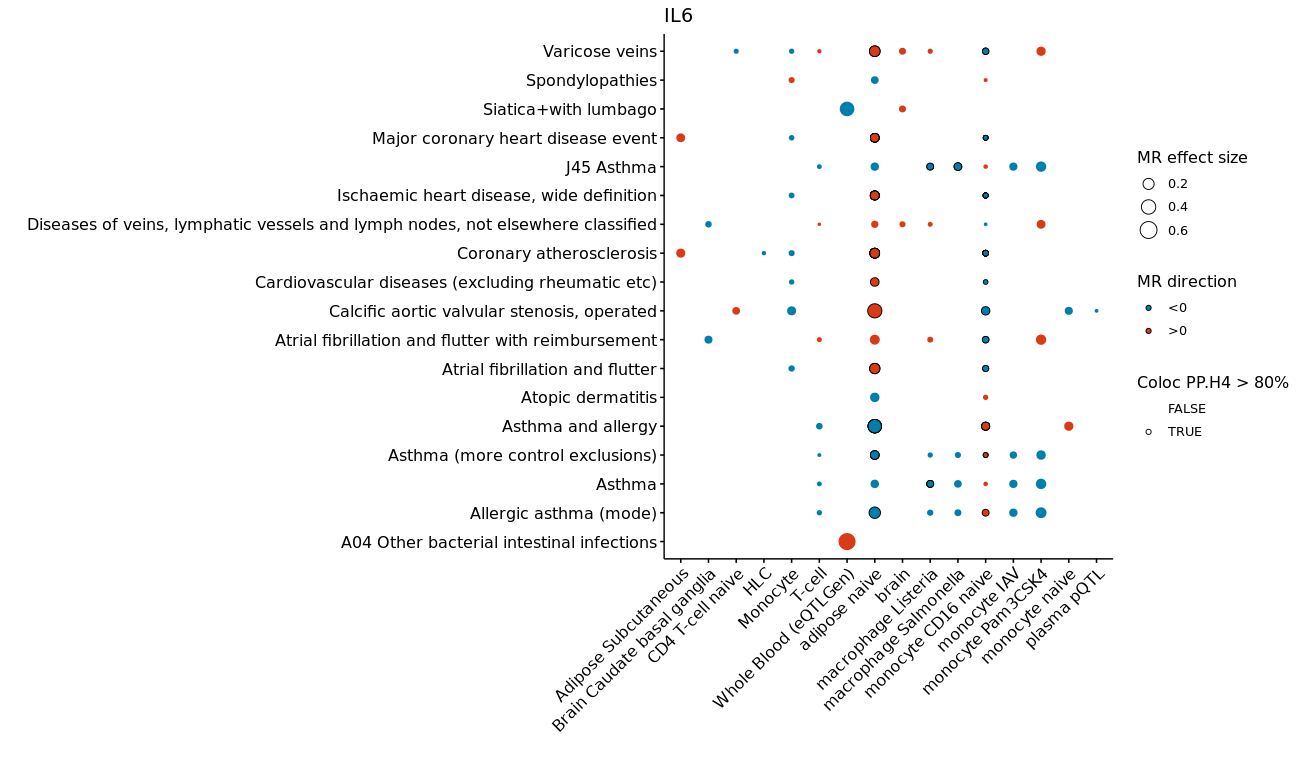
**

**Figure S9. Association between *IL6* and diseases, supported by MR, colocalization and ABC**. The figure shows tissues and cell-types with significant MR (q-value < 0.05) using *IL6* molQTL as exposure and diseases as outcome (red: positive effect size estimate [MR beta]; blue: negative effect size estimate). The size of the dots represents absolute MR effect size. Disease-molQTL pairs with a colocalization posterior probability > 80% are highlighted with a dark border.

**
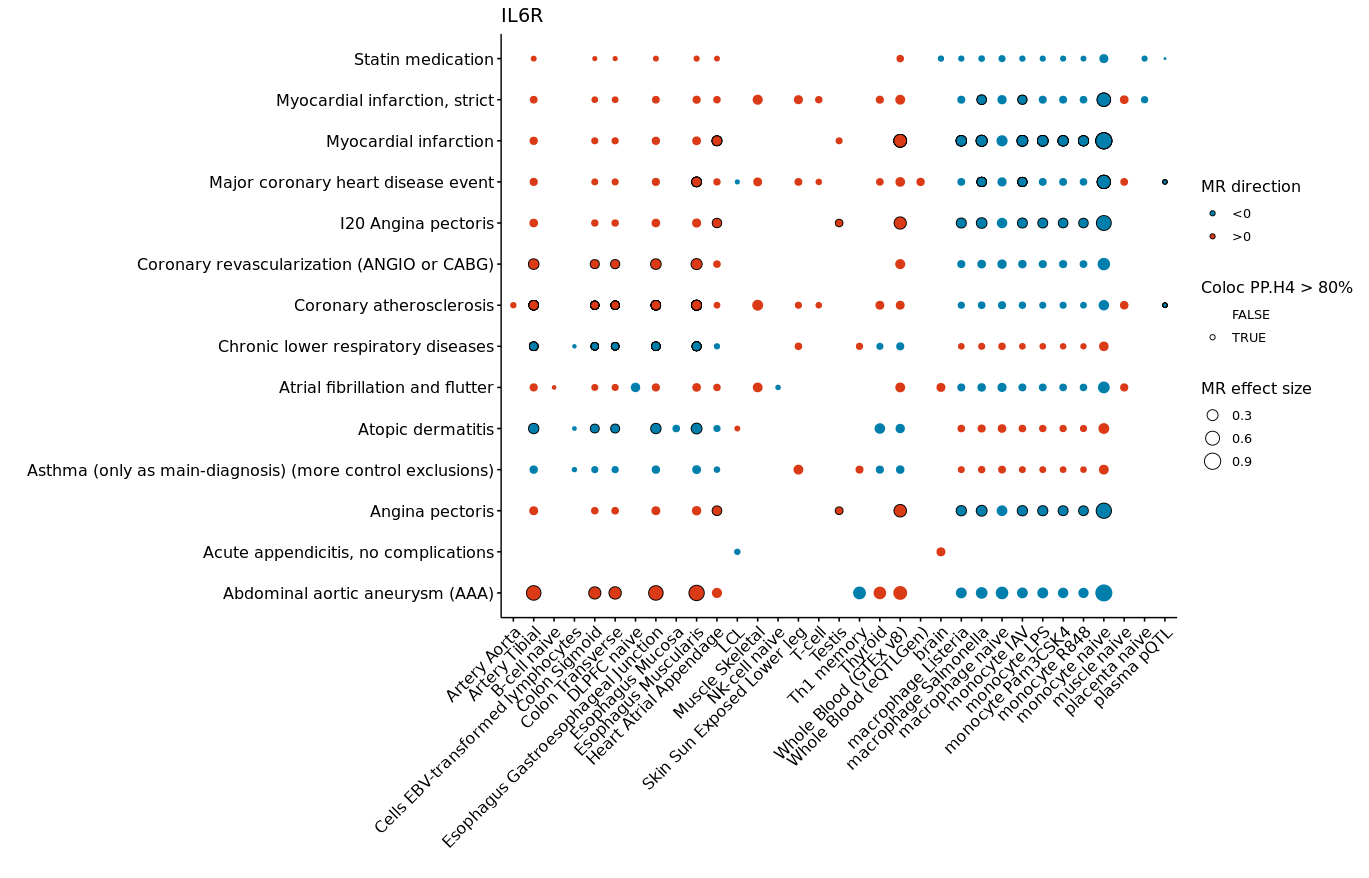
**

**Figure S10. Association between *IL6R* and diseases, supported by MR, colocalization and ABC**. The figure shows tissues and cell-types with significant MR (q-value < 0.05) using *IL6* molQTL as exposure and diseases as outcome (red: positive effect size estimate [MR beta]; blue: negative effect size estimate). The size of the dots represents absolute MR effect size. Disease-molQTL pairs with a colocalization posterior probability > 80% are highlighted with a dark border.

**REFERENCES**

1. Mountjoy E, Schmidt EM, Carmona M, Schwartzentruber J, Peat G, Miranda A, Fumis L, Hayhurst J, Buniello A, Karim MA, et al: **An open approach to systematically prioritize causal variants and genes at all published human GWAS trait-associated loci.** *Nat Genet* 2021, **53:**1527-1533.

2. Stein D, Bayrak ÇS, Wu Y, Stenson PD, Cooper DN, Schlessinger A, Itan Y: **Genome-wide prediction of pathogenic gain- and loss-of-function variants from ensemble learning of diverse feature set.** *bioRxiv* 2022.

3. McLaren W, Gil L, Hunt SE, Riat HS, Ritchie GR, Thormann A, Flicek P, Cunningham F: **The Ensembl Variant Effect Predictor.** *Genome Biol* 2016, **17:**122.

4. King EA, Dunbar F, Davis JW, Degner JF: **Estimating colocalization probability from limited summary statistics.** *BMC Bioinformatics* 2021, **22:**254.
